# Phragmites Communis (Lu Gen) Alleviates Hyperuricemia via Xanthine Oxidase Inhibition and Gut-Microbiota-Mediated Metabolite Urate Transporter Regulation

**DOI:** 10.3390/ph19091474

**Published:** 2026-09-17

**Authors:** Aamir Saleem, Bushra Walayat, Qidi Xue, Immad Ansari, Xiaoqing Wei, Ming Li

**Affiliations:** College of Basic Medical Science, Dalian Medical University, Dalian 116044, China; amirsaleem75000@outlook.com (A.S.); bushrawalayat55@outlook.com (B.W.); kidi_x@hotmail.com (Q.X.); immad.ansari@outlook.com (I.A.)

**Keywords:** hyperuricemia, hippuric acid, *Phragmites communis* rhizoma, uric acid transporters, xanthine oxidase

## Abstract

**Background:** Hyperuricemia (HU) results from excessive uric acid (UA) production/impaired excretion and is associated with gout and renal dysfunction. Gut microbiota dysbiosis may impair intestinal UA excretion, disrupt the gut barrier, and promote inflammation. Traditional Chinese medicine (TCM), with multitarget and microbiota-modulating effects, may offer a therapeutic strategy for HU. In this study, the rhizome of *Phragmites communis*, a TCM used for clearing heat and promoting diuresis, was investigated. Specifically, we evaluated whether *P. communis* could alleviate HU by suppressing xanthine oxidase (XOD) and modulating urate transporter expression, thereby reducing UA production while promoting its excretion. **Method:** The effects of a crude extract of *P. communis* were investigated with in vitro and in vivo models using molecular, 16S rRNA sequencing, and metabolomic analyses. **Results:** *P. communis* significantly decreased serum uric acid (SUA) levels by inhibiting XOD and improving renal and intestinal urate excretion, accompanied by decreased renal and increased intestinal solute carrier family 2 member 9 (SLC2A9) and enhanced ATP-binding cassette subfamily G member 2 (ABCG2) expression, restored gut barrier integrity, and suppressed inflammation. Microbiota analysis revealed enrichment of *Ligilactobacillus*, *Prevotella*, and *Dubosiella*. Fecal metabolomics identified hippuric acid (HA), which correlated with *Ligilactobacillus* abundance. In vitro, HA enhanced ABCG2, PDZ domain-containing 1 (PDZK1), and SLC2A9 expression. Molecular docking identified feruloylquinic acid (FAQ) as the favorable XOD inhibitor. **Conclusions:** *P. communis* exerts anti-hyperuricemic effects by inhibiting UA biosynthesis, promoting UA excretion, modulating gut microbiota, restoring gut barrier integrity, and suppressing inflammation, highlighting its potential as a TCM-based therapeutic intervention for HU.

## 1. Introduction

Hyperuricemia (HU) is a complex metabolic disease manifested by chronically elevated serum uric acid (UA) [1,2,3]. The human body synthesizes UA as an end product of purine metabolism [4,5]. Excessive dietary intake of purines and fructose leads to elevated UA. Consequently, HU is more prevalent in developed countries [6,7,8] and is expected to increase with global economic growth. HU is associated with several comorbidities, such as diabetes, hypertension, cardiovascular diseases, hyperlipidemia, and gout [9,10].

UA is the final product in purine metabolism generated by the enzyme xanthine oxidase (XOD) [1]. Increased XOD activity causes excessive UA biosynthesis and reactive oxygen species (ROS) production, which exacerbates oxidative stress and tissue injury [11]. Insufficient uric acid excretion accounts for nearly 90% of HU cases [12]. UA is excreted via intestinal and renal routes by specialized transporters. In the kidney, UA reabsorption is primarily mediated by GLUT9, whereas its excretion is mainly facilitated by ABCG2 [13,14]. GLUT9 and ABCG2 also contribute to intestinal UA excretion [15]. In contrast, PDZK1 is a scaffold protein that binds to several uric acid transporters, including ABCG2, and mediates their subcellular localization [12]. However, HU is often accompanied by kidney and intestinal injury [16], which alters the expression of these transporters, reduces UA excretion, and further elevates serum UA [17]. Consequently, UA transporters represent a critical therapeutic target.

Increasing evidence indicates that intestinal UA excretion is closely associated with gut microbiota dysbiosis [18]. UA from the cells enters the gut via epithelial cell transporters, where it is either directly excreted or degraded by the gut microbiota. However, in HU, the gut microbial ecosystem is disturbed, characterized by reduced beneficial microbial diversity, depletion of SCFAs producing bacteria, and enrichment of pro-inflammatory taxa [19,20]. Conversely, restoring gut microbial homeostasis enhances intestinal urate degradation, promotes UA excretion, and alleviates inflammation [21]. These studies suggest that gut microbiota modulation is a promising therapeutic strategy for HU.

Currently, medical interventions for HU primarily inhibit UA synthesis and downregulate reabsorption transporters [22]. However, these commercially available drugs often cause severe adverse effects in patients, restricting their long-term clinical use [22]. In this context, TCM has gained increasing attention as a potential source of therapeutic agents due to their multitargeted mode of action and safety. Accumulating evidence demonstrates that TCM and its bioactive constituents can reshape the gut microbiota composition by enriching SCFA-producing and urate-degrading bacteria, suppressing pro-inflammatory taxa, restoring intestinal barrier integrity, and regulating microbial metabolite levels [23].

*Phragmitis Rhizoma*, commonly known as “Lu Gen” in traditional Chinese medicine, refers to the fresh or dried rhizome of *Phragmites australis* subsp. *australis* (Cav.) Trin. ex Steud., with *Phragmites communis* Trin. recognized as a synonym, widely distributed globally on banks of rivers, lakes, ponds, and low wetlands, historically prescribed for fever, nausea, and urinary disorders, reflecting its anti-inflammatory, detoxifying, and diuretic properties [24,25]. Currently, several Chinese patent medicines containing *P. communis* have been approved and are widely used in clinical practice in China. These formulations include Yin Qiao San and Sangju Ganmao granules for respiratory illness [26]. Phytochemical studies have revealed 83 compounds from *P. communis* including various flavonoids, phenolic acids, sterols, vitamins, and polysaccharides with antioxidant, anti-inflammatory, hepatoprotective, and nephroprotective effects [27,28,29]. Comprehensive phytochemical profiling has further identified polysaccharides, organic acids, flavonoids, steroids, phenylpropanoids, terpenoids, and alkaloids as principal constituents of *P. communis* rhizoma, including specific compounds such as the phenolic acids p-coumaric acid and ferulic acid, the phenylpropanoid glycoside N-[2-(5-Hydroxy-1H-indol-3-yl) ethyl]-p-coumaramide, and the indole alkaloid moschamindole [26]. Beyond their antioxidant, anti-inflammatory, hepatoprotective, and nephroprotective activities noted above, these constituents have also been reported to exert antibacterial, immunomodulatory, hypoglycemic, and lipid-lowering effects, and to protect against chemotherapy-induced myelotoxicity and skin aging [25,30]. As HU is closely associated with oxidative stress, activation of inflammatory signaling, and impaired renal urate handling, the pharmacological profile of *P. communis* aligns with the key pathogenic mechanisms of HU. Moreover, its diuretic potential may facilitate renal and intestinal uric acid excretion, and its anti-inflammatory and antioxidant properties may mitigate uric-acid-induced tissue damage. Collectively, these findings suggest that *P. communis* may attenuate the effects of HU. Therefore, this study was designed to evaluate the therapeutic potential of *P. communis* and elucidate its underlying mechanisms of action.

## 2. Results

### 2.1. Chemical Characterization of the P. communis Crude Extract

**Figure 1:**
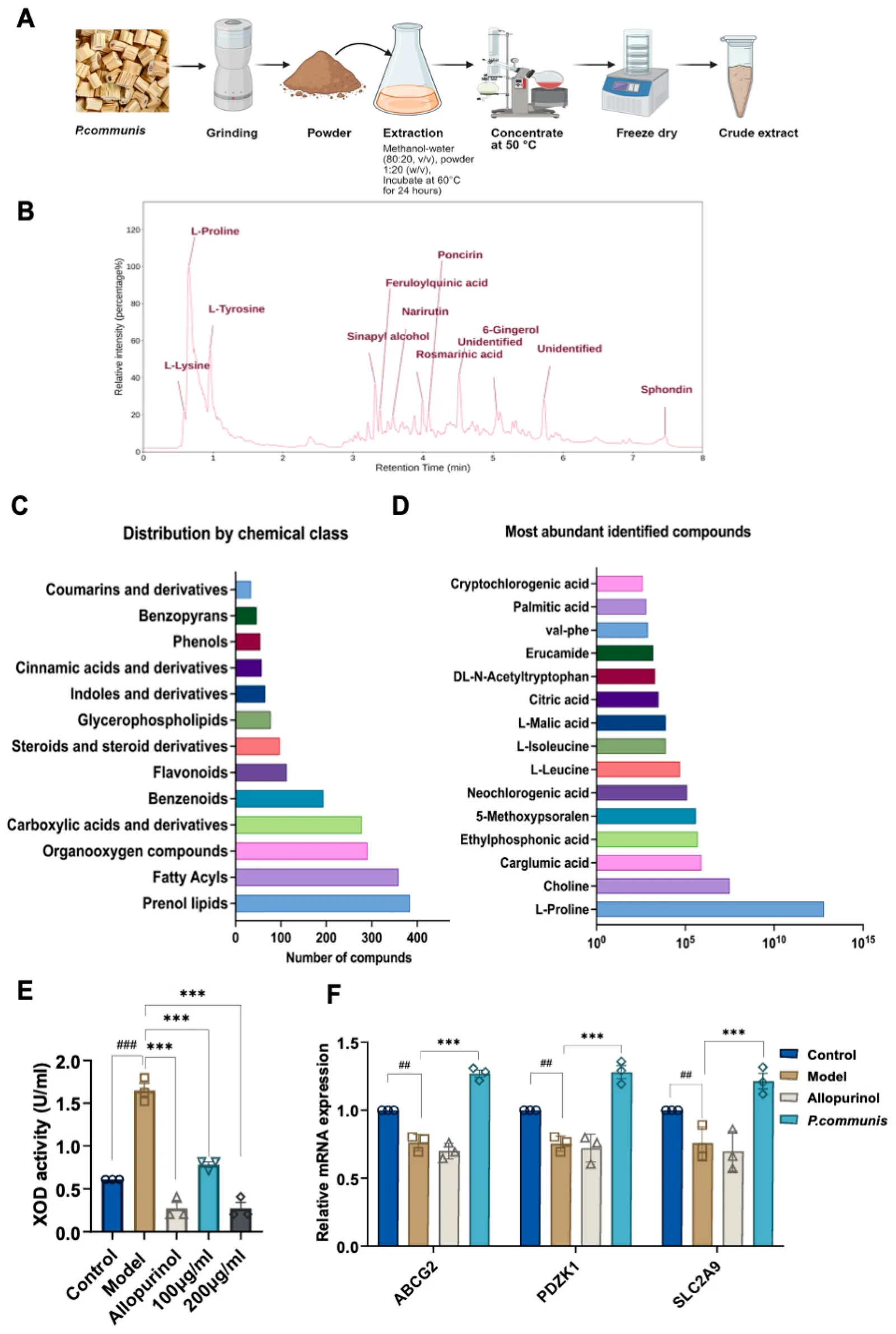
*P. communis* crude extract chemical characterization, and in vitro effects on uric acid metabolism. (**A**) Schematic representation of *P. communis* crude extract preparation. (**B**) Total ion chromatogram (TIC) of the crude extract with major constituents annotated. (**C**) Distribution of putatively annotated compounds by chemical class. (**D**) Most abundant putatively annotated constituents of the extract ranked by relative peak area. (**E**) In vitro XOD activity. (**F**) Relative expression of urate transporters, in vitro (**E**,**F**). *n* = 3, data represented ± SEM. Model vs. control, ## *p* < 0.01, and ### *p* < 0.001; model vs. *P. communis*, *** *p* < 0.001.

The extraction workflow for the *P. communis* rhizoma crude extract is shown in Figure 1A. UHPLC-MS profiling of the extract in positive and negative ionization modes putatively annotated 2105 and 1587 compounds, respectively, at a database match score ≥ 60 (Figure S1B,C). The putatively annotated compounds spanned a broad range of chemical classes, dominated by lipids and lipid-like molecules (23.8% in positive and 28.0% in negative mode), followed by organoheterocyclic compounds, organic acids and derivatives, benzenoids, phenylpropanoids and polyketides, and organic oxygen compounds (Figure S1B,C). The total ion chromatogram (TIC) was dominated by early-eluting free amino acids and organic acids, together with a cluster of moderately abundant peaks between 3 and 4.5 min corresponding mainly to phenolic acids and flavonoids, including feruloylquinic acid and rosmarinic acid (Figure 1B). The base peak chromatogram (BPC), which reflects the single most intense ion at each timepoint, was instead dominated by the furanocoumarin 5-methoxypsoralen (Figure S1A). At the class level, flavonoids, steroids and steroid derivatives, cinnamic acids and derivatives, phenols, and coumarins and derivatives, prenol lipids, fatty acyls, organooxygen compounds, carboxylic acids and derivatives, benzenoids, were prominently represented (Figure 1C).

Among the named, abundant constituents, the phenolic acids rosmarinic acid, neochlorogenic acid, and cryptochlorogenic acid, the furanocoumarin 5-methoxypsoralen, and free amino acids and organic acids (L-proline, choline, L-lysine, citric acid, and malic acid) were the most prominent. Compound identification confidence was assigned using a two-tier scheme: Level 1 annotations were confirmed against authentic reference standards analyzed under identical chromatographic and mass spectrometric conditions (retention time deviation ≤ 0.5 min, together with matching MS1 precursor and MS/MS fragmentation spectra), whereas Level 2 annotations were based on high-quality MS/MS spectral matching to database reference spectra (accurate mass and fragmentation pattern) in the absence of a corresponding standard. With the exception of 5-methoxypsoralen (Level 2, no standard available), all of the dominant constituents listed above, rosmarinic acid, neochlorogenic acid, cryptochlorogenic acid, L-proline, choline, L-lysine, citric acid, and malic acid, were confirmed against authentic reference standards (Level 1; Table S3).

Notably, the phenolic acid feruloylquinic acid was detected as an abundant, standard-confirmed (Level 1) constituent of the crude extract, alongside the related phenolic acids caffeic acid, ferulic acid, and chlorogenic acid isomers (neochlorogenic, cryptochlorogenic, and chlorogenic acid), all likewise confirmed against authentic standards (Figure 1D; Table S3).

### 2.2. P. communis Inhibits Xanthine Oxidase and Promotes UA Excretion Transporters In Vitro

Cell viability (HepG2, HT29 cells) was assessed using CCK8, and a non-toxic range of *P. communis* (200 µg/mL) was established for subsequent experiments (Figure S2A,B). To evaluate the effect of *P. communis* on uric acid metabolism, we first assessed the activity of the rate-limiting enzyme xanthine oxidase in vitro. Hypoxanthine treatment increased XOD activity compared to the control group, which was significantly inhibited by *P. communis* (Figure 1E), indicating the suppression of uric acid biosynthesis. Moreover, in HT29 cells mimicking HU, *P. communis* significantly enhanced the expression of uric acid excretion transporters SLC2A9 and ABCG2, as well as PDZK1(Figure 1F), whereas the positive control showed no significant effect on the transporters. These findings demonstrate that *P. communis* effectively modulates both uric acid production and excretion in vitro, supporting further evaluation in an animal model.

### 2.3. P. communis Ameliorates HU Parameters and Mitigates Systemic Inflammation in PO-AD-Induced HU Mice

**Figure 2:**
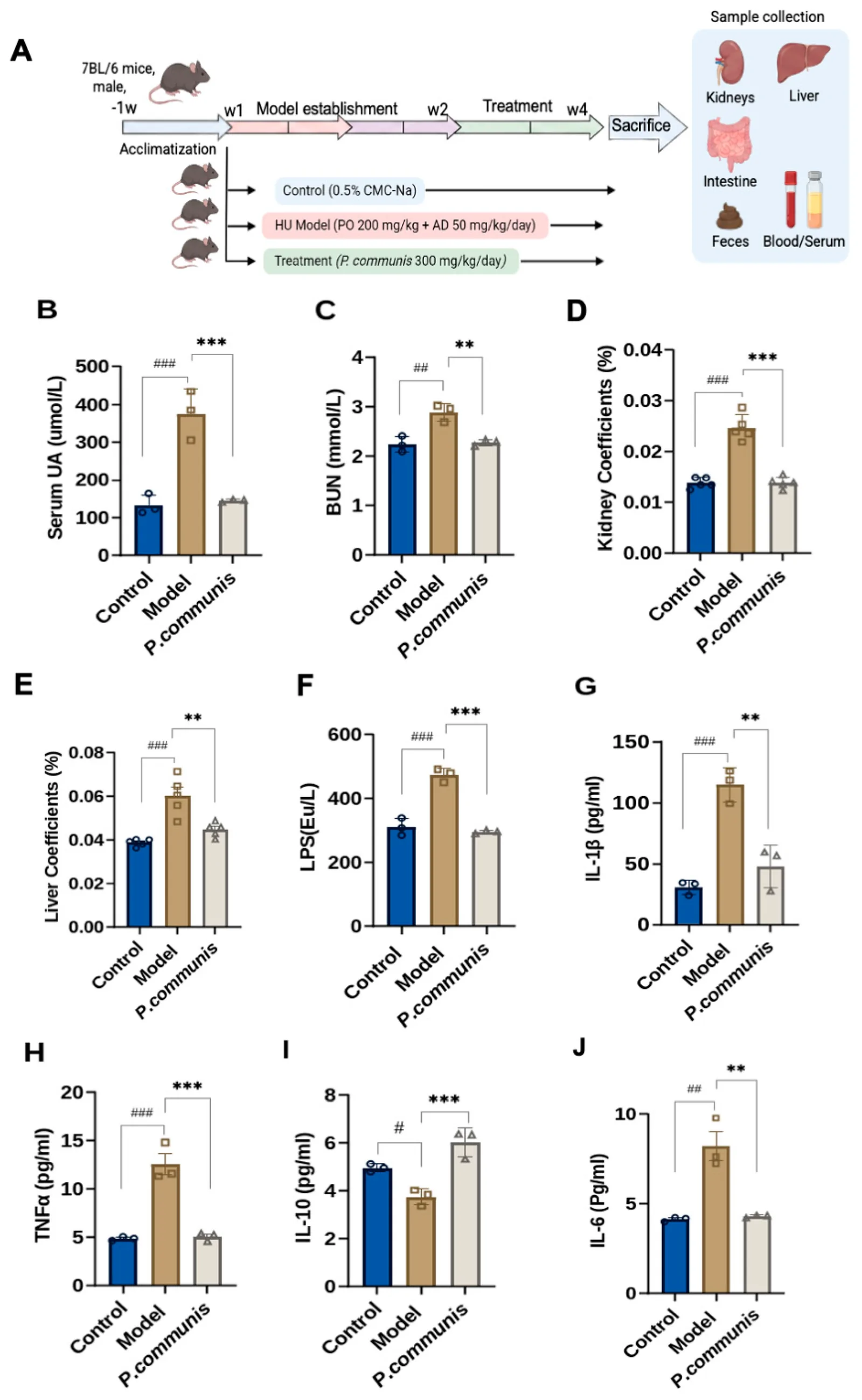
*P. communis* ameliorates biochemical and physiological parameters and mitigates systemic inflammation in PO-AD-induced HU mice. (**A**) In vivo experimental design; (**B**) serum uric acid level (SUA); (**C**) blood urea nitrogen level (BUN); (**D**) kidney coefficient; (**E**) liver coefficient, indicating the physiological improvement following *P. communis* administration; (**F**) serum LPS level; (**G**) serum IL-1β; (**H**) serum TNF-α; (**I**) serum IL-10; (**J**) serum IL-6 levels in different treatment groups. Sample sizes were *n* = 3 for panels (**B**,**C**,**F**–**J**), and *n* = 4 for panels (**D**,**E**). Data represented ± SEM. Model vs. control, # *p* < 0.05, ## *p* < 0.01, and ### *p* < 0.001; model vs. *P. communis*, ** *p* < 0.01, and *** *p* < 0.001.

The efficacy of *P. communis* was investigated in vivo using a hyperuricemic mouse model (Figure 2A). The hyperuricemia model group exhibited a significant reduction in body weight compared to the control group (Figure S3). SUA and BUN levels were elevated in the HU model, which were reversed to near-normal levels by *P. communis* (Figure 2B,C). The HU model group also exhibited significantly increased liver and kidney coefficients, reflecting organ stress (Figure 2D,E). These findings demonstrate that *P. communis* effectively alleviates HU in vivo.

Given that HU is closely associated with chronic inflammation, the effect of *P. communis* on the inflammatory response was assessed. The HU model exhibited elevated serum LPS and pro-inflammatory cytokines IL-1β, IL-6, and TNF-α, accompanied by decreased anti-inflammatory IL-10. *P. communis* mitigated these alterations by reducing LPS and pro-inflammatory cytokine levels and restoring IL-10 levels (Figure 2F–J). These findings indicate that *P. communis* effectively attenuates systemic and intestinal inflammation by regulating cytokine levels.

### 2.4. P. communis Regulates UA Biosynthesis and Enhances Its Excretion via Urate Transporters

**Figure 3:**
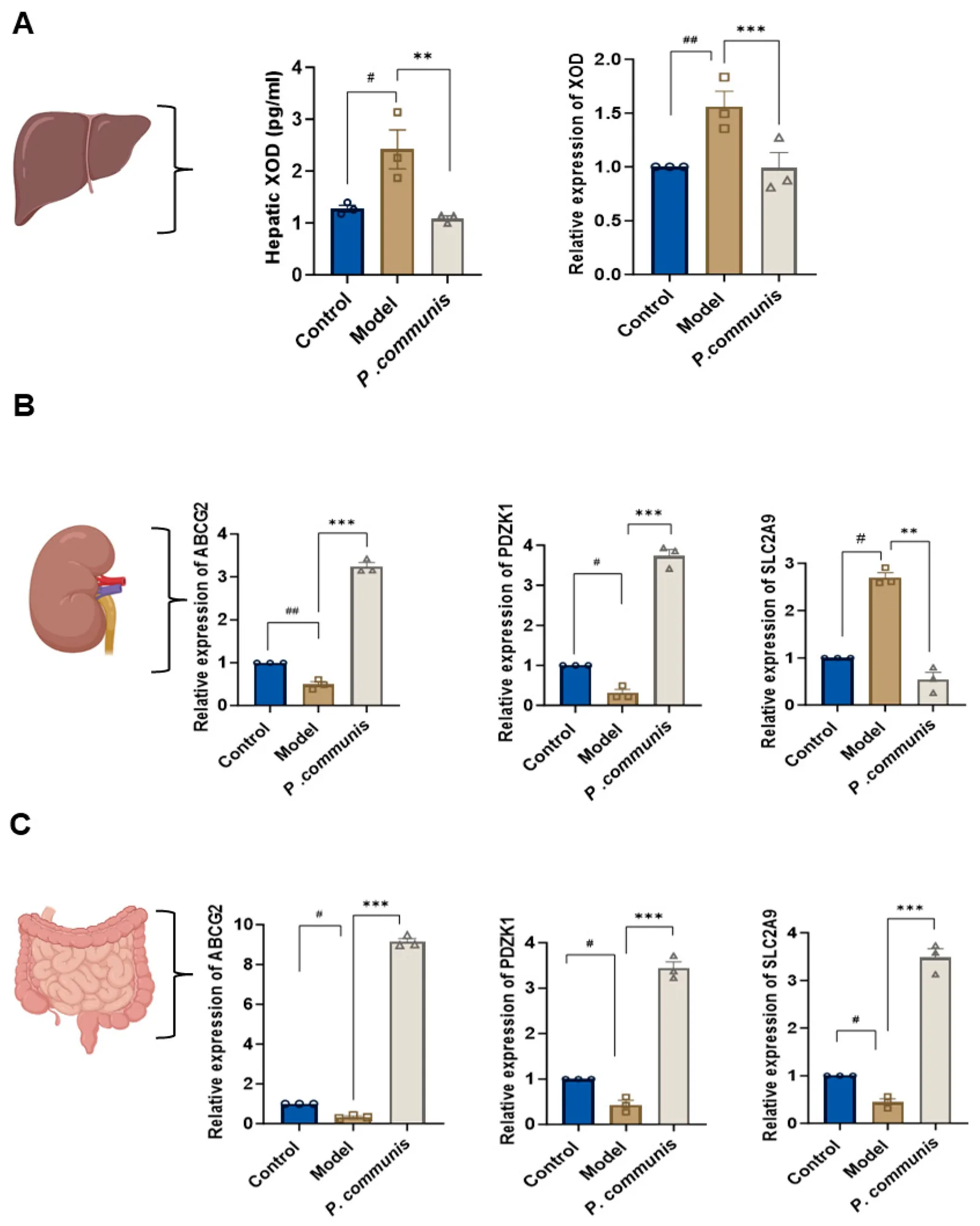
*P. communis* regulates UA biosynthesis and promotes renal and intestinal urate excretion via transporter modulation. (**A**) Liver xanthine oxidase (XOD) activity and mRNA expression. (**B**) Relative mRNA expression of renal transporters ABCG2, PDZK1, and SLC2A9. (**C**) Relative mRNA expression of intestinal transporters ABCG2, PDZK1, and SLC2A9. *n* = 3/group after exclusion of non-surviving mice during model induction (Section 4.7). Data represented ± SEM. Model vs. control, # *p* < 0.05, ## *p* < 0.01; model vs. *P. communis*, ** *p* < 0.01, and *** *p* < 0.001.

Given that UA production is catalyzed by XOD, hepatic XOD activity and mRNA expression were assessed. Notably, an increase in XOD activity and mRNA expression was observed in the HU model, which was significantly inhibited by *P. communis* treatment (Figure 3A).

Moreover, UA transporters in the kidneys and intestines were assessed. qPCR analysis revealed increased expression of SLC2A9, whereas the excretion transporter ABCG2 and its regulator PDZK1 were decreased in the model group, reflecting impaired renal uric acid excretion. *P. communis* intervention ameliorated these alterations by reducing SLC2A9 expression and enhancing ABCG2 and PDZK1 expression, thereby improving renal urate excretion (Figure 3B).

As the intestine is also an important extrarenal route for uric acid elimination, the expression of intestinal UA transporters was also assessed. The expression of SLC2A9 and ABCG2 was reduced in the model group, whereas *P. communis* administration significantly enhanced the expression of both SLC2A9 and ABCG2 (Figure 3C). These results indicate that *P. communis* restores systemic urate balance by inhibiting UA production and enhancing UA excretion.

### 2.5. P. communis Mitigates Inflammation and Restores Gut Barrier Integrity in PO-AD-Induced HU Mice

**Figure 4:**
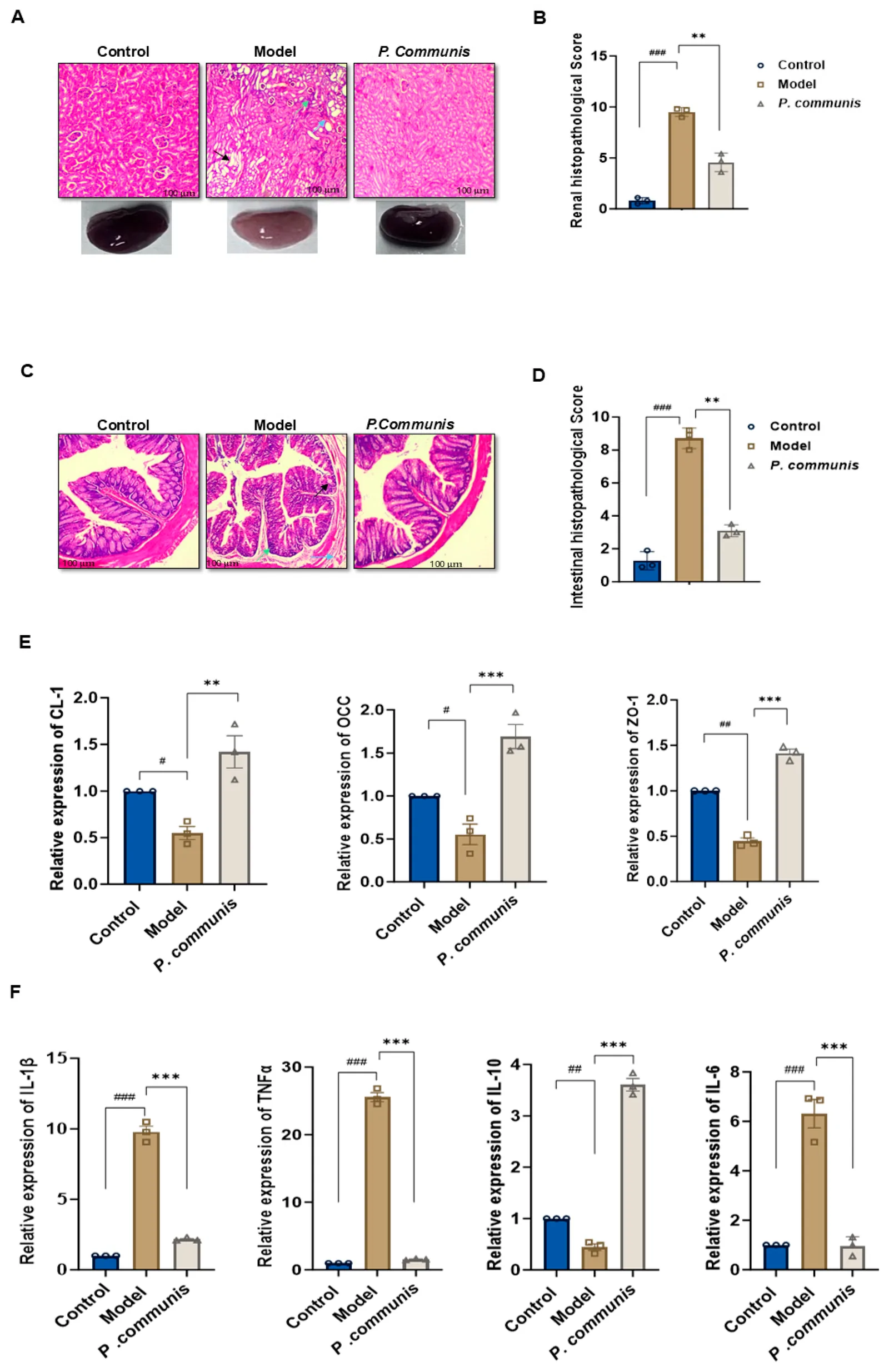
*P. communis* protects against renal and intestinal injury and restores intestinal barrier integrity in HU mice. (**A**) Representative H&E stain of renal tissue; green arrow indicates immune cell infiltration, blue arrow denotes hemorrhagic region, and black arrow highlights tubular dilation. (**B**) Histopathological score of renal injury. (**C**) H&E stain of intestinal tissue; green arrow denotes immune cell infiltration, black arrow denotes goblet cell disintegration, blue arrow denotes epithelial border disruption. (**D**) Histopathological score of intestine injury (scale bar = 200 μm, 10×). (**E**) Relative mRNA of intestinal junction proteins occluding (OCLN), claudin-1 (CLDN1), and zonula occludens-1 (ZO-1), showing restoration of barrier-associated gene expression in the treatment group. (**F**) Relative mRNA expression of IL-1β, TNF-α, IL-6, and IL-10 in intestinal tissues, showing the anti-inflammatory effects of *P. communis*. *n* = 3/group, data represented ± SEM. Model vs. control, # *p* < 0.05, ## *p* < 0.01, and ### *p* < 0.001; model vs. *P. communis*, ** *p* < 0.01, and *** *p* < 0.001.

Renal and intestinal injuries are critical contributors to HU progression and its systemic complications. The kidneys of the HU model group appeared pale and rough, whereas *P. communis* intervention restored a ruddy and smooth appearance (Figure 4A). Consistent with these findings, H&E staining demonstrated glomerular atrophy and tubular dilation in the HU model group, which was markedly alleviated by *P. communis* treatment (Figure 4A). Renal improvement was also verified by histopathological scores, assessed using the criteria in Table S2 (Figure 4B). In the intestine, HU caused villous atrophy, goblet cell rupture, and immune cell infiltration, which were markedly alleviated by *P. communis* treatment, as reflected in the intestinal histopathological score (Table S2) (Figure 4C,D). Consistently, the mRNA expression of intestinal tight junction proteins (ZO-1, occludin, and claudin-1) was significantly upregulated following *P. communis* intervention (Figure 4E). Moreover, mRNA expression of IL-1β, IL-6, and TNF-α in intestinal tissue was downregulated, whereas IL-10 expression was upregulated following *P. Communis* administration (Figure 4F). These findings demonstrate that *P. communis* protects the renal and intestinal structures and restores intestinal barrier integrity in HU mice.

### 2.6. P. communis Ameliorated Gut Microbiota Dysbiosis in PO-AD-Induced HU Mice

**Figure 5:**
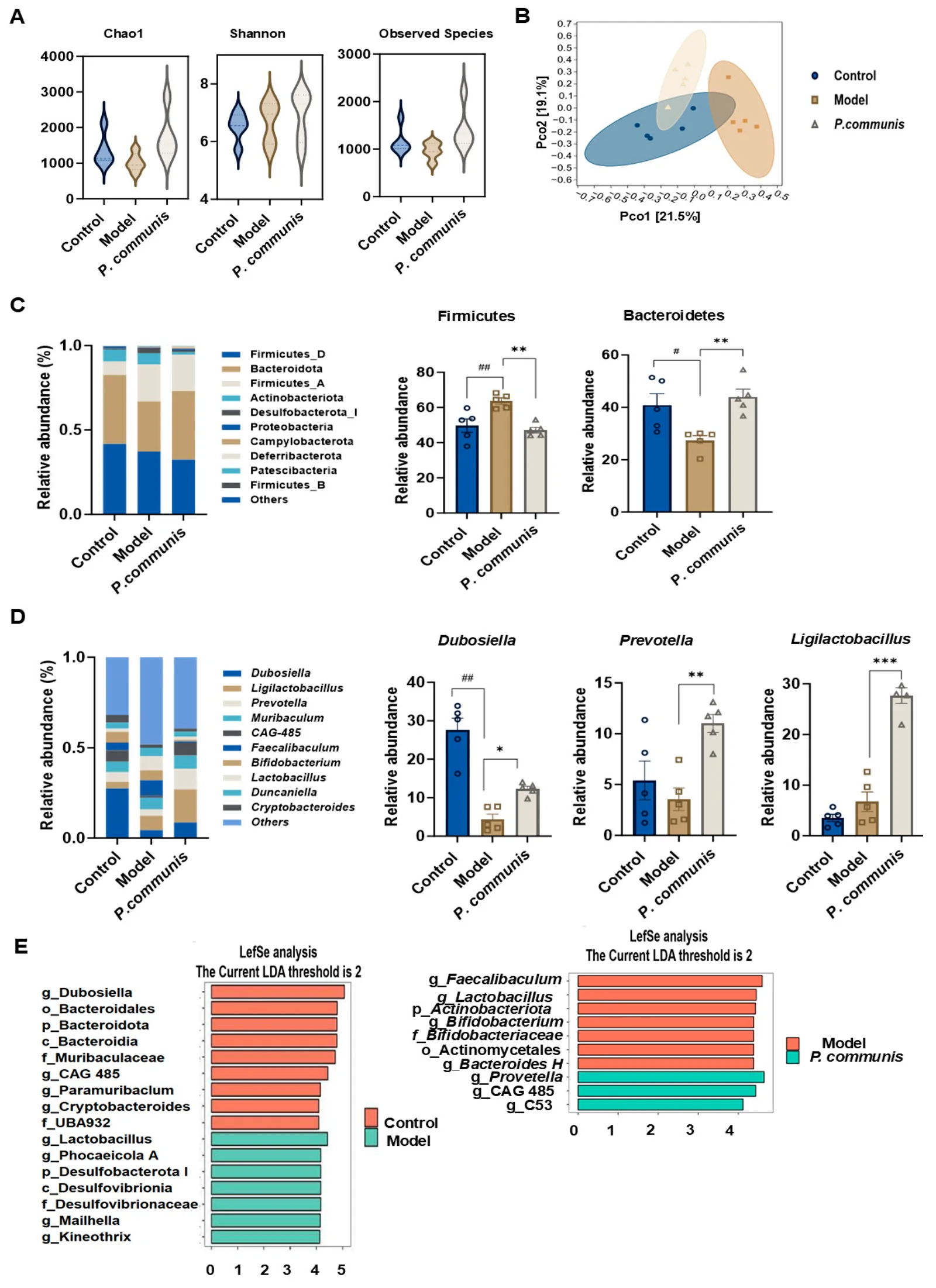
*P. communis* reshapes gut microbiota composition and diversity in PO-AD-induced HU. (**A**) Alpha diversity indices (Shannon, Chao1, and observed species) show microbial richness and diversity across groups. (**B**) Beta diversity analysis based on Bray–Curtis distance illustrating distinct microbial community clustering between groups. (**C**) Relative abundance at the phylum level highlights major bacterial shifts following *P. communis* treatment. (**D**) Relative abundance at the genus level, showing enrichment of key microbial taxa. (**E**) LEfSe analysis identifying discriminative bacterial biomarkers contributing to intergroup variation, *n* = 5. Data represented ± SEM. Model vs. control, # *p* < 0.05, ## *p* < 0.01; model vs. *P. communis*, * *p* < 0.05, ** *p* < 0.01, and *** *p* < 0.001.

Given the close association between gut dysbiosis and HU, we investigated whether *P. communis* exerted its protective effect by modulating the gut microbiota. For this purpose, 16S RNA sequencing was performed. The alpha diversity indices, including Chao1, Shannon, and observed species, were elevated in *P. communis* Rh, indicating increased microbial richness and diversity. Beta diversity analysis based on Bray–Curtis demonstrated a clear separation of the HU model group, whereas the control and *P. communis* group clustered closely, suggesting that *P. communis* restores the microbial diversity structure to normal (Figure 5A,B). At the phylum level, Bacteroidetes decreased in the HU model group but increased following *P. communis* treatment, indicating the restoration of the F/B ratio (Figure 5C). At the genus level, *Dubosiella* was reduced in the HU model group and increased after treatment, whereas *Ligilactobacillus* was significantly enriched in the treatment group. In addition, the abundance of the SCFA-producing bacteria *Prevotella* decreased in the model group but increased in the *P. communis* group, reflecting their contribution to a balanced gut microbiome (Figure 5D). LEfSe analysis identified *Dubosiella* and *Bifidobacterium* as key discriminative taxa enriched in the control group, with *Ruminococcus* and *Lachnospira* enriched in the *P. communis* group, supporting their protective and anti-inflammatory roles (Figure 5E). These microbial shifts suggest that *P. communis* modulates the gut microbiota composition, which may contribute to the mitigation of HU-related pathology.

### 2.7. Molecular Docking Identifies Feruloylquinic Acid as a Candidate Xanthine Oxidase Inhibitor

**Figure 6:**
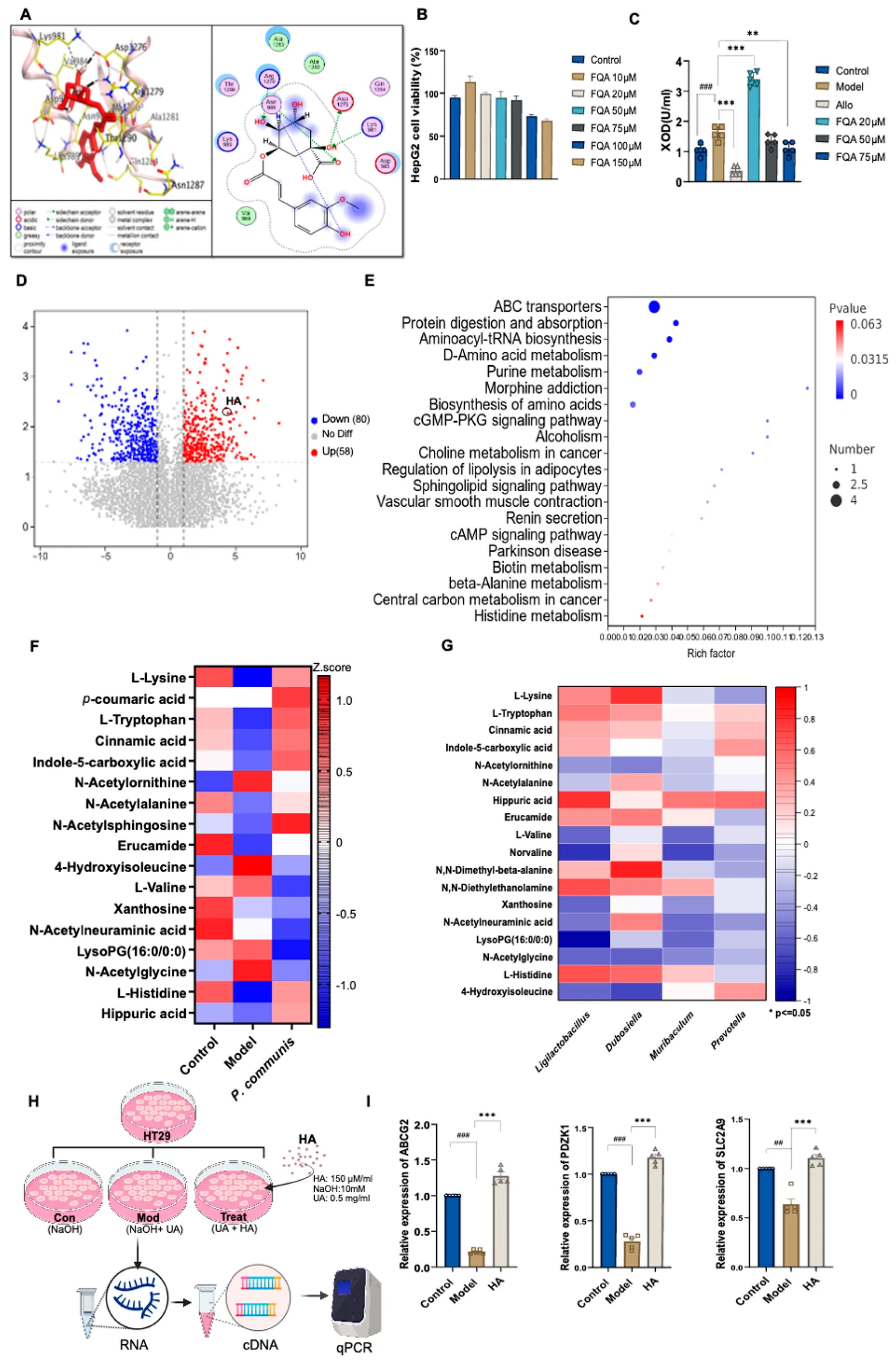
*P. communis* modulated molecular docking, metabolic profiles, and UA transporter expression in HT29 cells. (**A**) Molecular docking analysis (MOE; xanthine oxidase, PDB ID: 3NVW) of the 12 major phytochemicals tentatively identified in the crude extract, showing the top-ranked docking pose of feruloylquinic acid (FAQ) and its predicted interactions within the XOD active site. (**B**) HepG2 cell viability following treatment with FAQ (10–150 µM), establishing a non-toxic concentration range. (**C**) XOD activity in HepG2 cells treated with FAQ (20, 50, and 75 µM), confirming the docking prediction. (**D**) Volcano plot showing differential metabolites’ distribution between the model and *P. communis* group. (**E**) KEGG pathway enrichment analysis identifying key metabolic pathways affected by *P. communis* administration. (**F**) Heat map of altered metabolites across groups, including hippuric acid (HA). (**G**) Correlation analysis between altered fecal metabolites and gut microbial genera, highlighting the positive correlation between HA and *Ligilactobacillus*. (**H**) Schematic of the HT29 cell treatment workflow used for urate transporter gene expression analysis. (**I**) Relative mRNA expression of urate transporters (ABCG2, SLC2A9, and PDZK1) in HT29 cells following HA treatment. Fecal metabolomics analyses (**D**–**G**), *n* = 3/group; all other assays (**B**,**C**,**I**), *n* = 5/group. Data represented ± SEM. Model vs. control, ## *p* < 0.01, and ### *p* < 0.001; model vs. *P. communis*, ** *p* < 0.01, *** *p* < 0.001.

Having confirmed that the *P. communis* crude extract inhibits XOD both in vitro and in vivo, we next sought to identify which constituent phytochemicals might underlie this effect. Because phenolic acids and flavonoids are widely reported as potential XOD inhibitors, we hypothesized that the phenolic acid- and flavonoid-rich profile of the crude extract (Section 2.1) could account for its XOD-inhibitory activity. To test this, molecular docking was performed on the 12 major phytochemicals tentatively identified in the crude extract (UHPLC-MS identification score ≥ 80) against the crystal structure of xanthine oxidase (PDB ID: 3NVW) using MOE. Feruloylquinic acid (FAQ) showed the most favorable predicted binding affinity (docking score S = −6.03 kcal/mol), closely followed by rosmarinic acid (S = −6.02 kcal/mol), whereas the remaining compounds showed comparatively weaker predicted binding affinities (S ranging from −5.67 to −4.79 kcal/mol) (Table 1 and Table S4). Although the docking scores of FAQ and rosmarinic acid were nearly identical, FAQ exhibited a more extensive interaction network within the XOD binding site, involving multiple hydrogen-bond, side-chain, and backbone contacts with residues including Asn988, Lys981, Arg1279, and Asp1276, whereas rosmarinic acid showed comparatively fewer residue interactions, primarily involving Phe649 and Asp651 (Figure 6A,B). Based on this broader interaction profile, together with its detection in the *P. communis* extract by UHPLC-MS, and the comparatively limited characterization of FAQ as an anti-hyperuricemic candidate, FAQ was prioritized for subsequent experimental validation.

FAQ was selected for in vitro validation. A non-toxic concentration range of FAQ (10–150 µM) was first established in HepG2 cells by CCK-8 assay (Figure 6B). HepG2 cells were then treated with FAQ (20, 50, and 75 µM) followed by hypoxanthine induction, and XOD activity was measured. FAQ treatment significantly inhibited XOD activity in a concentration-dependent manner, consistent with the effect of the crude extract (Figure 6C). These findings indicate that FAQ is a key phytochemical contributor to the XOD-inhibitory activity of the *P. communis* crude extract.

### 2.8. Fecal Metabolomics and Correlation Analysis Identify Hippuric Acid as a Candidate Mediator of Urate Transporter Regulation

Since ABCG2, SLC2A9, and PDZK1 are expressed in the intestine and the gut microbiota was significantly altered by *P. communis* treatment (Section 2.6), we hypothesized that gut-derived metabolites might mediate the observed enhancement of intestinal urate transporters. To test this, fecal metabolomics analysis was performed to investigate metabolic alterations. OPLS-DA demonstrated a clear separation between the control and model groups (Figure S4). *P. communis* treatment upregulated 58 metabolites and downregulated 80 relative to the model group, with hippuric acid (HA) identified as one of the significantly altered metabolites (Figure 6D). KEGG analysis showed that ABC transporters were the most significantly enriched pathway, along with pathways related to protein digestion and amino acid metabolism (Figure 6E), and hierarchical clustering of the significantly altered metabolites is shown in Figure 6F.

To identify which altered metabolite might link the gut microbial changes to urate transporter regulation, correlation analysis was performed between the fecal metabolome and the 16S rRNA sequencing data. This analysis revealed a significant positive correlation between HA and the abundance of *Ligilactobacillus*, the genus most significantly enriched by *P. communis* treatment (Section 2.6) (Figure 6G). Based on this correlation, HA was selected as a candidate mediator for further cell-based experiments.

To assess the role of uric acid transporters, HT-29 cells were treated with HA (Figure S5) (Figure 6H), and SLC2A9, ABCG2, and PDZK1 mRNA expression levels were evaluated. Treatment with HA significantly upregulated ABCG2, PDZK1, and SLC2A9 expression (Figure 6I), providing a potential mechanistic link between the metabolomic changes and the urate-lowering effects of the crude extract.

## 3. Discussion

Dietary transition towards purine-, fructose-, and protein-rich foods has contributed to the increasing global prevalence of HU [11]. As conventional urate-lowering drugs are often associated with adverse effects, TCM has gained global attention owing to its favorable safety profile and multitargeted therapeutic potential [23,31,32]. Uric acid is produced mainly from purine metabolism, primarily catalyzed by XOD, and excessive XOD activity contributes to UA overproduction [33,34]. In this study, *P. communis* significantly inhibited liver XOD activity, demonstrating its effects on uric acid synthesis and homeostasis.

In addition to UA production, impaired UA excretion is a major HU contributor. The kidneys and intestines account for approximately 70% and 30% of UA excretion, respectively, and rely on specialized transporters for urate handling [35]. GLUT9, which is expressed on the apical and basolateral membranes of the renal proximal tubules, facilitates uric acid reabsorption. In contrast, ABCG2 facilitates the efflux of uric acid from renal tubules, and it has been reported that mice with defective ABCG2 or GLUT9 are more susceptible to hyperuricemia [36,37]. Recently, ABCG2 and GLUT9 have been reported to facilitate intestinal uric acid excretion [38]. Therefore, these transporters in the kidneys and intestines are important targets for the treatment of hyperuricemia [39]. However, few studies have reported TCM-based strategies for HU that simultaneously target both kidney and intestinal transporters. In this study, both the kidneys and intestines were considered, and *P. communis* was found to be effective in decreasing GLUT9 levels while enhancing ABCG2 and its modulator PDZK1 in the kidney. Moreover, *P. communis* effectively enhances excretion via ABCG2 and GLUT9 in the intestine. These results indicate that *P. communis* has multitarget effects on different organs.

*P. communis* exerts differential effects on renal uric acid transporters, consistent with prior studies showing that punicalagin inhibits GLUT9 in the kidney while enhancing its expression in the intestine [40]. Similarly, Estiverne et al. previously reported the differential impact of eurycomanol on GLUT9 expression in the kidney and intestine [41].

HU is closely associated with intestinal, renal injury, and chronic inflammation [40,42]. In the present study, HU mice exhibited elevated BUN levels, histopathological renal damage, and increased pro-inflammatory cytokine levels. All of these parameters were significantly alleviated by *P. communis* treatment, consistent with previous findings [40]. We observed a remarkable increase in the pro-inflammatory cytokines IL-1β, TNF-α, and IL-6. *P. communis* administration significantly decreased the pro-inflammatory cytokines as well as increased the anti-inflammatory cytokine IL-10. Renal and intestinal histopathology showed marked improvement, indicating effective attenuation of UA-induced tissue injury.

Accumulating evidence indicates that gut dysbiosis plays a central role in HU by disturbing purine metabolism, impairing intestinal urate degradation, and promoting systemic inflammation [18,40]. In this study, a significant increase in circulating LPS levels was observed in the HU model, along with impaired intestinal barrier integrity and reduced expression of TJPs. *P. communis* intervention reversed these changes by restoring TJPs and improving epithelial morphology, thereby reducing endotoxin translocation and systemic inflammation.

Moreover, *P. communis* markedly reshaped the gut microbiota by enriching beneficial genera. Notably, *Dubosiella*, known for its immunomodulatory effects and SCFA production that supports gut barrier integrity, was enriched [43,44]. *Prevotella*, which produces SCFAs to improve intestinal metabolism and barrier function, was also enriched [45]. *P. communis* treatment significantly increased the abundance of *Ligilactobacillus*, a genus reported to degrade purine and reduce uric acid levels [46,47]. The restoration of gut microbial balance by *P. communis* may further contribute to its therapeutic effects through microbiota-derived metabolites, including postbiotic compounds generated during probiotic–plant interactions; such metabolites may represent an additional mechanism underlying the beneficial effects of *P. communis* on gut barrier integrity, urate handling, and immune regulation, highlighting its potential as a plant-based intervention that promotes postbiotic-mediated benefits [48].

Metabolomic analysis revealed that HA was among the metabolites significantly altered in the HU model group and further modulated following *P. Communis* treatment. Correlation analysis between the fecal metabolome and 16S rRNA sequencing data revealed a significant positive correlation between HA and the abundance of *Ligilactobacillus*, the genus most significantly enriched by *P. communis* treatment, suggesting that HA production may be linked to the expansion of this beneficial taxon. HA is a well-characterized mammalian-microbial co-metabolite formed via hepatic glycine conjugation of microbially derived benzoate and related aromatic precursors, and its fecal and urinary abundance is widely used as a marker of gut microbial diversity and activity, being consistently associated with a diverse, healthy gut microbiota and reduced inflammatory tone [49]. Given that ABCG2 and GLUT9 are regulated not only by phytochemicals but also by changes in the metabolic environment [50,51], and given their correlation with *Ligilactobacillus*, HA was selected as a plausible candidate mediator linking the gut microbial shifts and in vivo metabolomic changes observed in this study to intestinal urate transporter regulation. To validate this, HT29 cells were treated with HA, which significantly enhanced ABCG2 expression, its regulator PDZK1, and SLC2A9, supporting a functional relationship between HA and intestinal urate transport. These findings suggest that HA may act as a gut-microbiota-derived modulator of intestinal urate transporters, providing a potential mechanistic bridge between the restoration of gut microbial balance and the urate-lowering effect of *P. communis*.

The chemical profile of the *P. communis* crude extract provides a chemical basis for the anti-hyperuricemic activity reported here. UHPLC-MS characterization tentatively identified a phenolic acid-, flavonoid-, and coumarin-rich profile, consistent with earlier phytochemical surveys of the genus Phragmites and earlier reports describing phenolic acids, including feruloylquinic acid (FAQ), caffeic acid, and ferulic acid, in the rhizoma of *P. communis* [52,53]. FAQ was directly detected in the crude extract as an abundant phenolic acid constituent (Table S3), and among the 12 major phytochemicals evaluated by molecular docking against the crystal structure of xanthine oxidase (MOE; PDB ID: 3NVW), it exhibited the most favorable binding affinity (S = −6.03 kcal/mol), narrowly ahead of rosmarinic acid (S = −6.02 kcal/mol) (Table 1). This docking prediction was confirmed in vitro, where FAQ significantly inhibited XOD activity (Section 2.7), supporting its role as a contributor to the XOD-inhibitory activity of the extract, consistent with kinetic and molecular simulation evidence that structurally related phenolic acids inhibit XOD [54]. The 75 µM concentration used to characterize XOD inhibition falls within the range reported for structurally related caffeoylquinic acids against this enzyme (IC50 ≈ 27–96 µM), and is well below the millimolar IC50 values reported for simpler phenolic acids on the same target [52]. MTT assays in HepG2 cells confirmed no significant loss of viability across the full tested range (10–150 µM) (Figure 6B), indicating that the observed inhibition is not attributable to cytotoxicity. Although peripheral plasma concentrations of FAQ after dietary intake are typically lower than 75 µM [54], xanthine oxidoreductase activity is concentrated in the intestinal mucosa and liver, tissues exposed to substantially higher local phenolic concentrations during absorption and first-pass metabolism than peripheral plasma reflects; the tested concentration is therefore both cellularly tolerable and physiologically plausible at the enzyme’s principal site of activity, with confirmation of systemic in vivo relevance to be addressed in future pharmacokinetic studies. The crude extract was also notably enriched in coumarins, with the furanocoumarin 5-methoxypsoralen identified as the single most abundant constituent of the base peak chromatogram (Figure S1A). Coumarin derivatives, including furanocoumarins, have been independently reported to act as xanthine oxidase inhibitors with concurrent free-radical-scavenging activity, providing a second, complementary phytochemical class through which *P. communis* may limit both uric acid biosynthesis and the oxidative stress that accompanies XOD catalysis [55]. Together, the convergence of phenolic acids and coumarins identified by UHPLC-MS with the observed inhibition of liver XOD activity provides a coherent phytochemical rationale for the urate-lowering effect of *P. communis*.

Several limitations should be noted. Although the dominant metabolites (feruloylquinic acid, rosmarinic acid, and related phenolic acids) were confirmed against authentic standards (Level 1; Table S3), their reported abundances are semi-quantitative peak-area estimates rather than absolute concentrations; therefore, their proportional contributions to the observed bioactivity cannot be determined from the present data. Future studies should quantify these metabolites. Similarly, the adenine/potassium oxonate-induced HU model relies on pharmacological uricase inhibition to compensate for rodents’ functional uricase and cannot fully reproduce human urate metabolism. In addition, HA and FAQ were validated only in vitro; future studies should evaluate purified HA and FAQ, alone and in combination, in vivo, including protein-level validation. Future in vivo studies should also include an appropriate pharmacological positive control to enable direct comparison with established urate-lowering therapies. The effective mouse dose (300 mg/kg/day for 14 days) corresponds to an estimated human equivalent dose (HED) of 24.3 mg/kg/day (~1.46 g/day for a 60 kg adult); however, this estimate does not account for interspecies differences in bioavailability, metabolism, or extract composition. Standardization, pharmacokinetic, safety, and dose-escalation studies are needed before clinical application.

## 4. Methods

### 4.1. P. communis Crude Extract Preparation

The dried *P. communis* rhizoma was purchased from Li Shizhen Pharmaceutical Group Co., Ltd., Huanggang, China, and the extract was prepared following the protocol described in a previous study [24]. Briefly, powder of the dried rhizoma of *Phragmites communis* (*P. communis*) was subjected to extraction using methanol-water (80:20, *v*/*v*) and incubated at 60 °C for 24 h. The mixture was then cooled and filtered, and the filtrate was concentrated using a rotary evaporator and freeze-dried to obtain a crude extract (Figure 1A).

### 4.2. Crude Extract Characterization by Ultra-High-Performance Liquid Chromatography–Mass Spectrometry (UHPLC-MS)

The chemical composition of the *P. communis* crude extract was characterized by ultra-high-performance liquid chromatography–mass spectrometry (UHPLC-MS). Approximately 50 mg of lyophilized extract was dissolved in pre-cooled methanol containing 5 ppm L-2-chlorophenylalanine as an internal standard, vortexed for 30 s, sonicated in an ice-water bath for 10 min, held at −20 °C for 30 min, and centrifuged at 12,000 rpm for 10 min at 4 °C. The supernatant was filtered through a 0.22 µm membrane before analysis.

Chromatographic separation was performed on an ACQUITY UPLC HSS T3 column (Waters Corporation, Milford, MA, USA) (100 Å, 1.8 µm, 2.1 × 100 mm) maintained at 40 °C with a flow rate of 0.4 mL/min. The mobile phases were 0.1% formic acid in water (A) and acetonitrile containing 0.1% formic acid (B). The gradient was 5% B from 0 to 1 min, increased linearly to 95% B from 1 to 4.7 min, held at 95% B from 4.7 to 6 min, returned to 5% B at 6.1 min, and re-equilibrated at 5% B from 6.1 to 8.5 min. The same gradient was used in positive and negative ionization modes.

Mass spectrometric detection was performed on a Thermo Orbitrap Exploris 120 equipped with a heated electrospray ionization (HESI) source (Thermo Fisher Scientific, Bremen, Germany), operated in both positive and negative modes using data-dependent acquisition (DDA). Spray voltages were 3.5 kV and −3.0 kV for positive and negative modes, respectively. Sheath and auxiliary gases were set to 40 and 10 arbitrary units, respectively; capillary and auxiliary gas heater temperatures were 320 °C and 300 °C, respectively. Full-scan MS1 spectra were acquired at 60,000 resolution over *m*/*z* 70–1000, with an automatic gain control (AGC) target of Standard and a maximum injection time of 100 ms. The four most intense precursors were selected for HCD fragmentation at 30% collision energy and analyzed at 15,000 resolution, with the AGC target set to Standard, maximum injection time set to Auto, and dynamic exclusion of 4 s.

Raw data were processed using MS-DIAL (version 4.9.221218) for peak extraction, alignment, filtering, and normalization. Peaks missing in >50% of samples were removed, and remaining missing values were imputed prior to normalization. A pooled quality-control (QC) sample, prepared by combining equal aliquots of all sample filtrates, was injected 2–4 times before the analytical sequence and every 6–12 sample injections thereafter to monitor system stability.

Compounds were putatively annotated by matching accurate mass and MS/MS spectra against the PSNGM (PerSonalbio Next-Generation Metabolomics Database), which incorporates mzCloud, LIPID MAPS, HMDB, MoNA, NIST 2020 MS/MS, an in-house authentic standards library, and an AI-predicted MS/MS library. Annotations with a score ≥ 60, an MS1 tolerance of 0.01 Da, and an MS2 tolerance of 0.05 Da were retained. Retained compounds were classified by chemical class, and peak areas were used to assess relative abundance and identify major constituents. The workflow followed the standardized protocol of Nanjing Personal Gene Technology Co., Ltd., Nanjing, China, without further method optimization for the sample matrix [56].

### 4.3. Assessment of Cell Viability Using Cell Counting Kit-8 (CCK-8) Assays

The viability of human colorectal adenocarcinoma (HT29) (TCH-C213, HyCyte, Suzhou, China) and human hepatocellular carcinoma (HepG2) (STCC10114P, Solarbio, Beijing, China) cell lines was assessed using CCK-8 assays (NCM Biotech, Suzhou, China). After the cells reached 80% confluence, they were seeded in a 96-well plate at a density of 1 × 10^4^ cells/well. The cells were exposed to *P. communis* crude extract at a concentration of 100–400 μg/mL for 24 h, and 10 μL of CCK-8 solution was added to each well. After incubation for one hour, the absorbance was measured at 450 nm.

### 4.4. Xanthine Oxidase (XOD) Inhibition Assay Using P. communis Crude Extract

HepG2 cells were cultured in DMEM containing 10% FBS and penicillin/streptomycin. After reaching ∼90% confluence, the cells were treated with *P. communis* crude extract (200 µg/mL) for 24 h, followed by exposure to 4 mM hypoxanthine to induce XOD activity. Allopurinol (100 µM) was used as a positive control. Xanthine oxidase activity was quantified using a commercial kit (Solarbio, Beijing, China) according to the manufacturer’s instructions.

### 4.5. Xanthine Oxidase (XOD) Inhibition Assay Using Feruloylquinic Acid

HepG2 cells were treated with feruloylquinic acid (FAQ; 20, 50, and 75 µM) for 24 h, followed by exposure to 4 mM hypoxanthine to induce XOD activity, using the same protocol and positive control described above for the crude extract.

### 4.6. Gene Expression Analysis of Uric Acid Transporters In Vitro

Given that the intestine contributes substantially to total urate elimination, and that plant-derived compounds are first metabolized within the intestinal environment where they may interact with the gut microbiota and intestinal epithelium, HT29 human colorectal epithelial cells were used to model the intestinal component of uric acid handling. HT29 cells were treated with 0.5 mg/mL uric acid in 10 mM NaOH and incubated for 24 h at 37 °C and 5% CO_2_ to mimic hyperuricemia. The cells were then exposed to the *P. communis* Rh crude extract. RNA was extracted using TRIzol reagent (Vazyme, Nanjing, China), reverse transcribed to cDNA (GenStar StarScript III RT MasterMix, Beijing, China), and the expression of uric acid transporter ABCG2, SLC2A9, and regulator PDZK1 was examined by qPCR. GAPDH served as the reference gene, and relative expression was calculated using the ∆∆Ct method. The primer sequences are listed in Table S1.

### 4.7. Establishment of a HU Mouse Model and Drug Administration

Male C57BL/6 mice (6 weeks old, 18–22 g) from Liaoning Changsheng Biotechnology Co., Ltd., Benxi, China (certificate number SYXK (Liao)2024–0002) were housed under specific pathogen-free (SPF) conditions (22–26 °C, 50% relative humidity, 12 h light/dark cycle) with ad libitum access to standard diet and water. Based on the in vitro results and preliminary dose assessment (100, 200, and 300 mg/kg (Figure S3A)), 300 mg/kg was selected for subsequent experiments. The HU model was established by oral administration of adenine (50 mg/kg) and potassium oxonate (200 mg/kg) for two weeks. Following successful HU model establishment, mice were randomly divided into *P. communis* treatment and HU model groups (*n* = 6). Mice in the *P. communis* treatment group were administered the *P. communis* crude extract orally at a dose of 300 mg/kg daily for two weeks [30]. After concluding the experiment, the mice underwent a 12 h fast and were then sacrificed. Blood, liver, kidney, and intestinal samples were collected and stored at −80 °C for subsequent analysis. Fecal samples were stored at −80 °C for 16S rRNA sequencing and metabolomic analysis.

### 4.8. Histopathological Examination of Renal and Intestinal Tissue

After dissection, kidney and intestinal tissues were fixed in 4% paraformaldehyde for 48 h, followed by dehydration using an ethanol gradient, embedding in paraffin, sectioning at 4 µm, and staining with hematoxylin and eosin (H&E). The sections were examined under a bright-field microscope at 200× and 400× magnification.

### 4.9. Organ Index

Organ index is commonly used as an indicator of organ hypertrophy, atrophy, or stress associated with disease progression or treatment. After sacrifice, the liver and kidneys were excised, rinsed in ice-cold saline, blotted dry, and weighed. The organ index was calculated as follows: (organ weight/body weight) × 100, with elevated values interpreted as indicative of organ stress or hypertrophy.

### 4.10. Biochemical Analysis

Blood samples were allowed to clot at room temperature and centrifuged at 3500 rpm for 15 min to obtain serum. Serum samples were then analyzed using ELISA kits (Mei Biao Biological Technology Co., Ltd., Yancheng, China). The measured parameters included uric acid, blood urea nitrogen (BUN), IL-1β, IL-6, TNF-α, IL-10, LPS, and xanthine oxidase, which were all determined according to the manufacturer’s instructions.

### 4.11. RNA Concentration and Quantification of mRNA Expression by Real-Time qPCR

Total RNA was extracted from the liver, kidney, and colon tissues using the RNA isolator Total RNA extraction reagent (Vazyme, Nanjing, China), following the manufacturer’s instructions. RNA purity and concentration were measured using a NanoDrop 2000 spectrophotometer (Thermo Fisher Scientific, Waltham, MA, USA). RNA samples were reverse-transcribed to cDNA using the StarScript III RT Mastermix (Genstar, Beijing, China). qPCR primers were designed using the Primerbank and ORIGENE databases, and their specificity was verified using NCBI Primer-BLAST (accessed in 2025). The expression levels of the uric acid transporters ABCG2, SLC2A9, PDZK1, TJP ZO-1, occludin, and claudin-1 were quantified using qPCR, with GAPDH as an internal reference. The primer sequences are listed in (Table S1).

### 4.12. 16S rRNA Sequencing Analysis

Microbial DNA was extracted from the fecal samples of mice using a stool DNA Kit (Omega Bio-Tek, Norcross, GA, USA) according to the manufacturer’s instructions. The quantity and quality of the extracted DNAs were measured using a NanoDrop NC2000 spectrophotometer (Thermo Fisher Scientific, Waltham, MA, USA) and agarose gel electrophoresis, respectively. The V3–V4 hypervariable region of the bacterial 16S rRNA was amplified using the universal primers 338F and 806R. The PCR products were purified, quantified, and used for library construction. Paired-end sequencing (2 × 250 bp) was performed using the Illumina Novaseq 6000 SP Reagent Kit (Shanghai Personal Biotechnology Co., Ltd., Shanghai, China). Microbiome bioinformatics were performed using QIIME2 2022.11. Raw reads were filtered, demultiplexed, and processed into amplicon sequence variants (ASVs). Taxonomic classification was performed using the SILVA 16S rRNA database. The microbial composition was summarized at different taxonomic levels, and relative abundance plots were generated at the family and genus levels of classification.

Alpha diversity was evaluated using the Shannon and Chao1 indices. Beta diversity was assessed using PCoA based on the Bray–Curtis index. The statistical significance of differences in community structure among groups was tested using PERMANOVA. Differentially abundant taxa were identified using Linear Discriminant Analysis Effect Size (LEfSe). Data visualization and statistical analyses were performed in R package (v4.2.0).

### 4.13. Non-Targeted Metabolomics Profiling of Mice Fecal Samples Using UPLC-MS

Metabolomic profiling of mouse fecal samples was performed using UHPLC-MS. Approximately 50 mg of fecal material was collected from each group before sacrifice and mixed with pre-cooled methanol (containing 5 ppm L-2-chlorophenylalanine), vortexed for 30 s, homogenized at 55 Hz for 30 s (twice), sonicated for 10 min, frozen at −20 °C for 30 min, and centrifuged at 12,000 rpm, 4 °C for 10 min. The supernatant was filtered through a 0.22 µm membrane for further analyses. Chromatographic separation was performed on an ACQUITY UPLC HSS T3 column (Waters Corporation, Milford, MA, USA; 100 Å, 1.8 µm, 2.1 × 100 mm) maintained at 40 °C with a flow rate of 0.4 mL/min. The mobile phases consisted of acetonitrile and 0.1% formic acid in the water. The elution gradient was 5% acetonitrile, which was linearly increased to 100% within 20 min and then returned to 5% in the final 2 min. Mass spectrometry was performed using a Thermo Orbitrap Exploris (Thermo Fisher Scientific, Bremen, Germany) 120 instrument equipped with HESI source (3.5 kV/3.0 kV). The raw data were processed using Compound Discoverer 3.3. Multivariate statistical PLS-DA analysis and multivariate statistical OPLS-DA analysis were performed using the R package Ropls, and KEGG pathways enrichment analysis was conducted using cluster Profiler (v4.6.0).

### 4.14. Molecular Docking

Molecular docking was performed to predict the binding affinities of the 12 major phytochemicals tentatively identified in the crude extract (selected on the basis of a UHPLC-MS identification score ≥ 80) for xanthine oxidase. Ligand structures of the 13 phytochemicals were retrieved from PubChem, and the three-dimensional crystal structure of xanthine oxidase (PDB ID: 3NVW) was retrieved from the RCSB Protein Data Bank (https://www.rcsb.org/structure/3NVW) accessed on 1 March 2026. Protein preparation was performed using QuickPrep in Molecular Operating Environment (MOE 2022; Chemical Computing Group), including protonation at physiological pH (7.0), correction of structural inconsistencies, removal of distant water molecules, and restrained energy minimization. The binding site was defined using the Ligand Atom mode based on the coordinates of the co-crystallized ligand, guanine. Docking was performed using the Triangle Matcher placement method with the default MOE scoring functions. To validate the docking protocol, guanine was redocked into the XOD binding pocket, and the ability of the method to reproduce the native binding pose was confirmed. Following validation, the 13 phytochemicals were docked into the XOD active site using the same parameters, yielding the final docking score (S, kcal/mol), with more negative values indicating more favorable predicted binding.

### 4.15. Cell Viability and UA Transporters Gene Expression Analysis for Evaluation of the Hippuric Acid (HA) Effect

HT29 cell viability was assessed using the CCK-8 assay (NCM Biotech is located in Suzhou, China). Cells at 80% confluence were seeded in 96-well plates at a density of 1 × 10^4^ cells per well and treated with HA (10, 50, 100, 150, 200 and 300 µM) for 24 h. The cells were then incubated with 10 µL of CCK-8 reagent for 1 h at 37 °C. Absorbance was measured at 450 nm to determine the cell viability.

For transporter expression analysis, HT29 cells were treated with 500 µM uric acid dissolved in 10 mM NaOH and incubated for 24 h at 37 °C and 5% CO_2_ to mimic hyperuricemia conditions, followed by exposure to 150 µM HA. Total RNA was extracted using TRIzol reagent (Vazyme, Nanjing, China). The expression levels of ABCG2, SLC2A9, and PDZK1 were quantified using qPCR, with GAPDH as an internal control. Relative expression levels were calculated using the ∆∆Ct method.

### 4.16. Statistical Data Analysis

All statistical analyses were performed using GraphPad Prism software (version 10). Data are presented as mean ± SEM (*n* ≥ 3). Group means were compared using ordinary one-way analysis of variance (ANOVA) followed by Dunnett’s post hoc test, with the model group designated as the reference group for comparisons with the treatment groups. Homogeneity of variance was assessed using the Brown–Forsythe test, and the normality of residuals was formally tested. No non-parametric tests were used. Statistical significance was defined as * *p* < 0.05, ** *p* < 0.01, and *** *p* < 0.001.

16S rRNA sequencing data were processed and analyzed using QIIME2 (version 2022.11). Metabolomics data were analyzed using multivariate statistical approaches, including partial least squares discriminant analysis (PLS-DA) and orthogonal partial least squares discriminant analysis (OPLS-DA), using the R package. For metabolite-level statistical comparisons, Student’s *t*-test was used for two-group comparisons, whereas one-way ANOVA was used for comparisons involving three or more groups. The resulting *p* values were corrected for multiple comparisons using the Benjamini–Hochberg (BH) procedure, and the adjusted values are reported as false discovery rate (FDR) or q-values. The FDR-adjusted q-values were used to control for multiple hypothesis testing in the metabolomics analyses.

## 5. Conclusions

Collectively, these findings indicate that *P. communis* reduced serum UA levels, alleviated renal and intestinal injury, improved gut barrier integrity, modulated UA transporters, and restored gut microbial homeostasis, with HA and FAQ emerging as potential contributors to its urate-lowering effects. Future studies should focus on clarifying how FAQ inhibits xanthine oxidase (XOD) activity and how HA enhance urate excretion through relevant transporters. In addition, their therapeutic efficacy and mechanistic relevance should be validated in well-controlled in vivo hyperuricaemia models, including appropriate positive-control treatments, to establish their pharmacological potential and translational relevance. These studies will further clarify whether HA and FAQ represent key mediators of the anti-hyperuricaemic effects of *P. communis*.

## Figures and Tables

**Table 1:** Molecular docking results (MOE; xanthine oxidase, PDB ID: 3NVW) for the phenolic acid, flavonoid, and coumarin constituents of the *P. communis* crude extract selected for docking (UHPLC-MS identification score ≥ 80), ranked by identification score. Feruloylquinic acid (bold) exhibited the most favorable docking score and was selected for in vitro validation (Section 2.7). The full list of compounds putatively annotated in the crude extract by UHPLC-MS, including free amino acids and sterols/vitamins, is provided in Table S3.

Rank	Compound	Category	UHPLC-MS Score	PubChem CID	Docking Score S (kcal/mol)
1	Homoeriodictyol	Flavonoids and glycosides	98.4	73635	−5.65
2	trans-Caffeic acid	Phenolic acids	98.4	689043	−5.44
3	Tyrosol	Phenolic acids	97.9	10393	−5.27
4	Sinapyl alcohol	Phenolic acids	96.5	5280507	−5.48
5	Rosmarinic acid	Phenolic acids	96.0	5281792	−6.02
**6**	**Feruloylquinic acid**	**Phenolic acids**	**94.3**	**10133609**	**−6.03**
7	trans-4-Coumaric acid	Phenolic acids	93.2	637542	−4.79
8	Aesculin	Coumarins and derivatives	92.8	5281417	−5.67
9	Sphondin	Coumarins and derivatives	92.4	108104	−5.14
10	Wogonin	Flavonoids and glycosides	92.3	5281703	−5.47
11	Dihydrobiochanin A	Flavonoids and glycosides	91.0	439784	−5.31
12	Scopoletin	Coumarins and derivatives	90.7	5280460	−5.36

Note: Docking Score (S) values are from molecular docking performed in Molecular Operating Environment (MOE) against the crystal structure of xanthine oxidase (PDB ID: 3NVW); more negative values indicate more favorable predicted binding.

## Data Availability

The original contributions presented in this study are included in the article/Supplementary Materials. Further inquiries can be directed to the corresponding author.

## Supplementary Materials

The following supporting information can be downloaded at: https://www.mdpi.com/article/10.3390/ph19091474/s1, Figure S1: Chemical characterization of the *P. communis* crude extract by UHPLC-MS. (A) Base peak chromatogram (BPC) with additional identified constituents annotated. (B) Distribution of putatively annotated compounds by chemical class in negative ionization mode (NEG). (C) Distribution of putatively annotated compounds by chemical class in positive ionization mode (POS); Figure S2: In vitro safety profile of the *P. communis* crude extract. (A) HT29 cell viability following treatment with *P. communis* crude extract at increasing concentrations (100–400 µg/mL). (B) HepG2 cell viability following treatment with *P. communis* crude extract at increasing concentrations (100–400 µg/mL), determined by CCK-8 assay; Figure S3: (A) Serum uric acid (SUA) concentrations were measured in the Control, Model, and *P. communis* crude extract groups (100, 200, and 300 mg/kg). Data are presented as mean ± SEM; * *p* < 0.05, ** *p* < 0.01, and *** *p* < 0.001 versus the Model group. (B) Changes in body weight over time for the Control, HU model, and *P. communis* treatment groups in the main hyperuricemic mouse model. Values are represented as mean ± SEM; *** *p* < 0.001; (C) Distribution of putatively annotated metabolities by chemical class in positive ionization mode (POS); Figure S4: OPLS-DA score plot showing separation among the control and model groups; Figure S5: HT29 cell viability following treatment with hippuric acid (HA) at increasing concentrations (50–300 µM), determined by CCK-8 assay; Table S1: Primer sequences used for qPCR; Table S2: Histopathological scoring criteria used for evaluation of intestinal and renal tissue injury (related to Figure 4B,D); Table S3: Full list of compounds identified in the *P. communis* crude extract by UHPLC-MS, including phenolic acids, flavonoids, coumarins, free amino acids, and sterols/vitamins (related to Section 3); Table S4: Full molecular docking output (MOE) for the 13 phytochemicals evaluated against xanthine oxidase (PDB ID: 3NVW), including docking score (S), RMSD, and MOE energy terms (E_conf, E_place, E_score1, E_refine, E_score2) for each docking pose.

## Abbreviations

HU: hyperuricemia; UA, uric acid; SUA, serum uric acid; BUN, blood urea nitrogen; ABCG2, ATP-binding cassette superfamily G member 2; SLC2A9, Solute Carrier Family 2 member 9; PDZK1, PDZ domain-containing 1; XOD, xanthine oxidase; H&E, hematoxylin and eosin; SCFA, short-chain fatty acid; CMC-Na, sodium carboxymethyl cellulose; PO, potassium oxonate; AD, adenine; HA, hippuric acid; FAQ, feruloylquinic acid; Rh, rhizoma; mzCloud, mzCloud Mass Spectral Database; LIPID MAPS, Lipid Metabolites and Pathways Strategy; HMDB, Human Metabolome Database; MoNA, MassBank of North America; NIST 2020, National Institute of Standards and Technology 2020 Mass Spectral Library; KEGG, Kyoto Encyclopedia of Genes and Genomes; TCM, Traditional Chinese Medicine; HED, Human Equivalent Dose.

## References

[1] Yanai H., Adachi H., Hakoshima M., Katsuyama H. (2021). Molecular Biological and Clinical Understanding of the Pathophysiology and Treatments of Hyperuricemia and Its Association with Metabolic Syndrome, Cardiovascular Diseases and Chronic Kidney Disease. Int. J. Mol. Sci..

[2] Sui X., Church T.S., Meriwether R.A., Lobelo F., Blair S.N. (2008). Uric Acid and the Development of Metabolic Syndrome in Women and Men. Metabolism.

[3] Bardin T., Richette P. (2014). Definition of Hyperuricemia and Gouty Conditions. Curr. Opin. Rheumatol..

[4] El Ridi R., Tallima H. (2017). Physiological Functions and Pathogenic Potential of Uric Acid: A Review. J. Adv. Res..

[5] Choi H.K., Atkinson K., Karlson E.W., Willett W., Curhan G. (2004). Purine-Rich Foods, Dairy and Protein Intake, and the Risk of Gout in Men. N. Engl. J. Med..

[6] Dehlin M., Jacobsson L., Roddy E. (2020). Global Epidemiology of Gout: Prevalence, Incidence, Treatment Patterns and Risk Factors. Nat. Rev. Rheumatol..

[7] Hisatome I., Li P., Miake J., Taufiq F., Mahati E., Maharani N., Utami S.B., Kuwabara M., Bahrudin U., Ninomiya H. (2021). Uric Acid as a Risk Factor for Chronic Kidney Disease and Cardiovascular Disease―Japanese Guideline on the Management of Asymptomatic Hyperuricemia. Circ. J..

[8] Li Y., Shen Z., Zhu B., Zhang H., Zhang X., Ding X. (2021). Demographic, Regional and Temporal Trends of Hyperuricemia Epidemics in Mainland China from 2000 to 2019: A Systematic Review and Meta-Analysis. Glob. Health Action.

[9] Amiya E. (2021). Link between Hyperuricemia, Renal Dysfunction, and Hypertension. J. Clin. Hypertens..

[10] Liu H., Xie R., Dai Q., Fang J., Xu Y., Li B. (2023). Exploring the Mechanism Underlying Hyperuricemia Using Comprehensive Research on Multi-Omics. Sci. Rep..

[11] Du L., Zong Y., Li H., Wang Q., Xie L., Yang B., Pang Y., Zhang C., Zhong Z., Gao J. (2024). Hyperuricemia and Its Related Diseases: Mechanisms and Advances in Therapy. Signal Transduct. Target. Ther..

[12] Mehmood A., Zhao L., Ishaq M., Xin W., Zhao L., Wang C., Hossen I., Zhang H., Lian Y., Xu M. (2020). Anti-Hyperuricemic Potential of Stevia (*Stevia Rebaudiana* Bertoni) Residue Extract in Hyperuricemic Mice. Food Funct..

[13] Sun H.L., Wu Y.W., Bian H.G., Yang H., Wang H., Meng X.M., Jin J. (2021). Function of Uric Acid Transporters and Their Inhibitors in Hyperuricaemia. Front. Pharmacol..

[14] Zhang S., Wang J., Wang S., Dai Z., Zhang L., Xue F., Heinbockel T. (2024). Role of Transporters in Hyperuricemia. Cell Communication and Signaling in Health and Disease.

[15] Xu X., Li C., Zhou P., Jiang T. (2016). Uric Acid Transporters Hiding in the Intestine. Pharm. Biol..

[16] Lu Y., Chang Y., Li T., Han F., Li C., Li X., Xue M., Cheng Y., Meng Z., Han Z. (2020). Empagliflozin Attenuates Hyperuricemia by Upregulation of ABCG2 via AMPK/AKT/CREB Signaling Pathway in Type 2 Diabetic Mice. Int. J. Biol. Sci..

[17] Sun L., Liu Q., Zhang Y., Xue M., Yan H., Qiu X., Tian Y., Zhang H., Liang H. (2023). Fucoidan from Saccharina Japonica Alleviates Hyperuricemia-Induced Renal Fibrosis through Inhibiting the JAK2/STAT3 Signaling Pathway. J. Agric. Food Chem..

[18] Bian M., Wang J., Wang Y., Nie A., Zhu C., Sun Z., Zhou Z., Zhang B. (2020). Chicory Ameliorates Hyperuricemia via Modulating Gut Microbiota and Alleviating LPS/TLR4 Axis in Quail. Biomed. Pharmacother..

[19] Singh A.K., Kumar Durairajan S.S., Durairajan S.S.K., Iyaswamy A., Williams L.L. (2024). Elucidating the Role of Gut Microbiota Dysbiosis in Hyperuricemia and Gout: Insights and Therapeutic Strategies. World J. Gastroenterol..

[20] Lee Y., Werlinger P., Suh J.W., Cheng J. (2022). Potential Probiotic Lacticaseibacillus Paracasei MJM60396 Prevents Hyperuricemia in a Multiple Way by Absorbing Purine, Suppressing Xanthine Oxidase and Regulating Urate Excretion in Mice. Microorganisms.

[21] Pan L., Han P., Ma S., Peng R., Wang C., Kong W., Cong L., Fu J., Zhang Z., Yu H. (2020). Abnormal Metabolism of Gut Microbiota Reveals the Possible Molecular Mechanism of Nephropathy Induced by Hyperuricemia. Acta Pharm. Sin. B.

[22] Wang J., Chen Y., Zhong H., Chen F., Regenstein J., Hu X., Cai L., Feng F. (2022). The Gut Microbiota as a Target to Control Hyperuricemia Pathogenesis: Potential Mechanisms and Therapeutic Strategies. Crit. Rev. Food Sci. Nutr..

[23] Azevedo V.F., Buiar P.G., Giovanella L.H., Severo C.R., Carvalho M. (2014). Allopurinol, Benzbromarone, or a Combination in Treating Patients with Gout: Analysis of a Series of Outpatients. Int. J. Rheumatol..

[24] Huang Z., Li B., Bi M.H. (2025). Advances in Traditional Chinese Medicine for the Treatment of Hyperuricemia and Associated Diseases: Pathogenesis, Mechanisms, and Future Directions. Front. Nutr..

[25] Ha C.W., Kim S.H., Lee S.R., Jang S., Namkoong S., Hong S., Lim H., Kim Y.K., Sohn E.-H. (2020). Anti-Skin Aging Potential of Alcoholic Extract of Phragmites Communis Rhizoma. Korean J. Plant Resour..

[26] Ren Y., Cui G.D., He L.S., Yao H., Zi C.Y., Gao Y.X. (2022). Traditional Uses, Phytochemistry, Pharmacology and Toxicology of Rhizoma Phragmitis: A Narrative Review. Chin. J. Integr. Med..

[27] Yun K.W., Seo K. (2023). Comparison of in vitro Antioxidant Capacities of Phragmites Communis Trin. And Phragmites Japonica Steud. Korean J. Food Preserv..

[28] In M., Oh N., Kim D.C. (2022). Nitrite Scavenging and Lipid Peroxidation Inhibitory Effects of Solvent Fractions from *Phragmites Communis* rhizoma Extract. J. Appl. Biol. Chem..

[29] Kang K.-Y., Park K.-W. (2025). Suppression of LPS-Induced Inflammation by Phragmites Communis Young Leaf Extract via Multi-Target Inhibition of IκB, AP-1, and STAT1/3 Pathways in RAW 264.7 Cells. Plants.

[30] Kim J., Lee Y.J., Kim Y.A., Cho E.S., Huh E., Bang O.S., Kim N.S. (2017). Aqueous Extract of Phragmitis Rhizoma Ameliorates Myelotoxicity of Docetaxel in vitro and in vivo. BMC Complement. Altern. Med..

[31] Bai H., Zhang Z., Zhu M., Sun Y., Wang Y., Li B., Wang Q., Kuang H. (2024). Research Progress of Treating Hyperuricemia in Rats and Mice with Traditional Chinese Medicine. Front. Pharmacol..

[32] Yang L., Wang B., Ma L., Fu P. (2022). Traditional Chinese Herbs and Natural Products in Hyperuricemia-Induced Chronic Kidney Disease. Front. Pharmacol..

[33] Rao L., Dong B., Chen Y., Liao J., Wang C., Fu G., Wan Y. (2024). Study on the Mechanism of Lactic Acid Bacteria and Their Fermentation Broth in Alleviating Hyperuricemia Based on Metabolomics and Gut Microbiota. Front. Nutr..

[34] Lee Y., Kim S., Yuk H.J., Kim D.-S. (2018). DKB114, a Mixture of Chrysanthemum Indicum Linne Flower and Cinnamomum Cassia (L.) J. Presl Bark Extracts, Improves Hyperuricemia Through Inhibition of Xanthine Oxidase Activity and Increasing Urine Excretion. Nutrients.

[35] Brovold H., Lund T., Svistounov D., Solbu M.D., Jenssen T.G., Ytrehus K., Zykova S.N. (2019). Crystallized but Not Soluble Uric Acid Elicits Pro-Inflammatory Response in Short-Term Whole Blood Cultures from Healthy Men. Sci. Rep..

[36] Adomako E.A., Moe O.W. (2023). Uric Acid Transport, Transporters, and Their Pharmacological Targeting. Acta Physiol..

[37] Toyoda Y., Mančíková A., Krylov V., Morimoto K., Pavelcová K., Bohatá J., Pavelká K., Pavlíková M., Suzuki H., Matsuo H. (2019). Functional Characterization of Clinically-Relevant Rare Variants in ABCG2 Identified in a Gout and Hyperuricemia Cohort. Cells.

[38] Ichida K., Matsuo H., Takada T., Nakayama A., Murakami K., Shimizu T., Yamanashi Y., Kasuga H., Nakashima H., Nakamura T. (2012). Decreased Extra-Renal Urate Excretion Is a Common Cause of Hyperuricemia. Nat. Commun..

[39] Zhang M.-Q., Sun K.-X., Guo X., Chen Y.-Y., Feng C.-Y., Chen J.-S., Barreira J.C.M., Prieto M.A., Sun J.-Y., Zhang J.-D. (2023). The Antihyperuricemia Activity of Astragali Radix through Regulating the Expression of Uric Acid Transporters via PI3K/Akt Signalling Pathway. J. Ethnopharmacol..

[40] Han Q.Q., Ren Q.D., Guo X., Farag M.A., Zhang Y.H., Zhang M.Q., Chen Y.Y., Sun S.T., Sun J.Y., Li N.Y. (2024). Punicalagin Attenuates Hyperuricemia via Restoring Hyperuricemia-Induced Renal and Intestinal Dysfunctions. J. Adv. Res..

[41] Bao R., Chen Q., Li Z., Wang D., Wu Y., Liu M., Zhang Y., Wang T. (2022). Eurycomanol Alleviates Hyperuricemia by Promoting Uric Acid Excretion and Reducing Purine Synthesis. Phytomedicine.

[42] Isaka Y., Takabatake Y., Takahashi A., Saitoh T., Yoshimori T. (2016). Hyperuricemia-Induced Inflammasome and Kidney Diseases. Nephrol. Dial. Transplant..

[43] Li B., Qiao Y., Li Y. (2025). Porphyromonas Gingivalis Deteriorates Autism Spectrum Disorders by Disturbing the Gut and Oral Microbiota. Front. Microbiol..

[44] Hankel J., Sander S., Muthukumarasamy U., Strowig T., Kamphues J., Jung K., Visscher C. (2022). Microbiota of Vaccinated and Non-Vaccinated Clinically Inconspicuous and Conspicuous Piglets Under Natural Lawsonia Intracellularis Infection. Front. Vet. Sci..

[45] Rodríguez J.M., Garranzo M., Segura J., Orgaz B., Arroyo R., Alba C., Beltrán D., Fernández L. (2023). A Randomized Pilot Trial Assessing the Reduction of Gout Episodes in Hyperuricemic Patients by Oral Administration of Ligilactobacillus Salivarius CECT 30632, a Strain With the Ability to Degrade Purines. Front. Microbiol..

[46] Ma N., Cai S., Sun Y., Chu C. (2024). Chinese Sumac (Rhus Chinensis Mill.) Fruits Prevent Hyperuricemia and Uric Acid Nephropathy in Mice Fed a High-Purine Yeast Diet. Nutrients.

[47] Baran M., Váradyová Z., Kráčmár S., Hedvábný J. (2002). The Common Reed (Phragmites Australis) as a Source of Roughage in Ruminant Nutrition. Acta Vet. Brno.

[48] Al-Habsi N., Al-Khalili M., Haque S.A., Elias M., Al Olqi N., Al Uraimi T. (2024). Health Benefits of Prebiotics, Probiotics, Synbiotics, and Postbiotics. Nutrients.

[49] Lees H.J., Swann J.R., Wilson I.D., Nicholson J.K., Holmes E. (2013). Hippurate: The Natural History of a Mammalian-Microbial Cometabolite. J. Proteome Res..

[50] Narayanan S., Gujarati N.A., Wang J., Wu Z., Koya J., Cui Q., Korlipara V.L., Ashby C.R., Chen Z. (2021). The Novel Benzamide Derivative, VKNG-2, Restores the Efficacy of Chemotherapeutic Drugs in Colon Cancer Cell Lines by Inhibiting the ABCG2 Transporter. Int. J. Mol. Sci..

[51] Takabe K., Kim R.H., Allegood J.C., Mitra P., Ramachandran S., Nagahashi M., Harikumar K.B., Hait N.C., Milstien S., Spiegel S. (2010). Estradiol Induces Export of Sphingosine 1-Phosphate from Breast Cancer Cells via ABCC1 and ABCG2. J. Biol. Chem..

[52] Farouk O.Y., Fahim J.R., Attia E.Z., Kamel M.S. (2023). Phytochemical and Biological Profiles of the Genus *Phragmites* (Family Poaceae): A Review. S. Afr. J. Bot..

[53] Gao H.-X., Ding A.-W., Tang Y.-P., Zhang X., Duan J.-A. (2009). Chemical Constituents from the Rhizomas of *Phragmites communis*. Chin. J. Nat. Med..

[54] Wang X., Su D., Luo X., Chen B., Bhullar K.S., Liu H., Wang C., Zhang J., Wang L., Yang H. (2025). Inhibition of Xanthine Oxidase by Four Phenolic Acids: Kinetic, Spectroscopic, Molecular Simulation, and Cellular Insights. Foods.

[55] Saekhor K., Udomsinprasert W., Honsawek S., Tachaboonyakiat W. (2019). Preparation of an injectable modified chitosan-based hydrogel approaching for bone tissue engineering. Int. J. Biol. Macromol..

[56] Huang X., Yang X., Xu C., Liu J., Luo Y., Xu Z., Pu S., Li Z., Zhang Y., Bai M. (2026). From Reshaped Metabolome to Repaired Skin: Fermented *Gastrodia elata* Alleviates UVB-Induced Damage through Controlled Immune Activation. Antioxidants.

