# Supplementary material for: Phragmites Communis (Lu Gen) Alleviates Hyperuricemia via Xanthine Oxidase Inhibition and Gut-Microbiota-Mediated Metabolite Urate Transporter Regulation
Source: Pharmaceuticals (Basel). 2026 Sep 17;19(9):1474. doi: 10.3390/ph19091474 (PMC13610185; doi:10.3390/ph19091474)
Supplement: Supplementary file 1 [file pharmaceuticals-19-01474-s001.zip › Table_S1.pdf]

Table S1. Primer Sequences Used for qPCR

Gene	Primer	Sequence (5'-3')	Source
**SLC2A9**	F	GCCATCATTGCCTCGTTCTGCA	Mouse
**SLC2A9**	R	TACGGCGAAGTTTGAGAGCCAG	Mouse
**ABCG2**	F	GAACTCCAGAGCCGTTAGGAC	Mouse
**ABCG2**	R	CAGAATAGCATTAAGGCCAGGTT	Mouse
**GAPDH**	F	AGGTCGGTGTGAACGGATTTG	Mouse
**GAPDH**	R	TGTAGACCATGTAGTTGAGGTCA	Mouse
**PDZK1**	F	TTGGCCTTCAGTCTGCTCG	Mouse
**PDZK1**	R	CAGAAGTGGTTGACGGCGAA	Mouse
**IL1-β**	F	CCACAGACCTTCCAGGAGAATG	Mouse
**IL1-β**	R	GTGCAGTTCAGTGATCGTACAGG	Mouse
**IL-6**	F	TACCACTTCACAAGTCGGAGGC	Mouse
**IL-6**	R	CTGCAAGTGCATCATCGTTGTTC	Mouse
**IL-10**	F	CGGGAAGACAATAACTGCACCC	Mouse
**IL-10**	R	CGGTTAGCAGTATGTTGTCCAGC	Mouse
**TNF-α**	F	GGTGCCTATGTCTCAGCCTCTT	Mouse
**TNF-α**	R	GCCATAGAACTGATGAGAGGGAG	Mouse
**ZO-1**	F	GTTGGTACGGTGCCCTGAAAGA	Mouse
**ZO-1**	R	GCTGACAGGTAGGACAGACGAT	Mouse
**CLD1**	F	GTCTTTGACTCCTTGCTGAATCTG	Mouse
**CLD1**	R	CACCTCATCGTCTTCCAAGCAC	Mouse
**Ocl**	F	TGGCAAGCGATCATACCCAGAG	Mouse
**Ocl**	R	CTGCCTGAAGTCATCCACACTC	
**GAPDH**	F	CATCACTGCCACCCAGAAGACTG	Mouse
**GAPDH**	R	ATGCCAGTGAGCTTCCCGTTCAG	Mouse
**ABCG2**	F	GTTCTCAGCAGCTCTTCGGCTT	Human
**ABCG2**	R	TCCTCCAGACACACCACGGATA	Human
**PDZK1**	F	AAGGTGTGGCTATGAGAGCTGG	Human
**PDZK1**	R	CAGCAGGAACATGACACGGCTT	Human
**SLC2A9**	F	GCTCTTGGAGAAGCACAACGAG	Human
**SLC2A9**	R	ACACCAGGCGGATGCTCCTCT	Human
**GAPDH**	F	GCCATCAAGGAGGCTGTAAAAGC	Human
	R	GGTATCACCGAGGAAGTCCGTA	Human
