# Supplementary material for: Phragmites Communis (Lu Gen) Alleviates Hyperuricemia via Xanthine Oxidase Inhibition and Gut-Microbiota-Mediated Metabolite Urate Transporter Regulation
Source: Pharmaceuticals (Basel). 2026 Sep 17;19(9):1474. doi: 10.3390/ph19091474 (PMC13610185; doi:10.3390/ph19091474)
Supplement: Supplementary file 1 [file pharmaceuticals-19-01474-s001.zip › Table_S3_updated.pdf]

Table S3. Full list of compounds identified in the Phragmites communis crude extract by UHPLC-MS, including phenolic acids, flavonoids, coumarins, free amino acids, and sterols/vitamins (related to Section 3).

S.No	Compound	RT (min)	m/z	Formula	Score	Mode	Database ID	Level	Rel. Abundance (×10⁶)
**Phenolic acids**									
1	*Rosmarinic acid*	3.91	359.0762	C18H16O8	96.0	NEG	C01850	Level 1	824.14
2	*Caffeic acid*	3.50	179.0349	C9H8O4	95.1	NEG	C01197	Level 1	777.03
3	*trans-Caffeic acid*	3.07	163.0388	C9H8O4	98.4	POS	C01197	Level 1	77.38
4	*Ferulic acid*	3.86	195.0653	C10H10O4	94.4	POS	C01494	Level 1	79.36
5	*trans-4-Coumaric acid*	3.77	165.0544	C9H8O3	93.2	POS	C00811	Level 1	49.51
6	*p-Coumaric acid glucoside*	3.11	371.0973	C15H18O8	92.8	NEG	C04415	Level 2	12.99
7	*Neochlorogenic acid*	3.33	353.0866	C16H18O9	90.8	NEG	C17147	Level 1	5,117.42
8	*Cryptochlorogenic acid*	3.33	355.1028	C16H18O9	93.0	POS	HMDB0030653	Level 1	2,601.92
9	*Feruloylquinic acid*	3.39	367.1037	C17H20O9	94.3	NEG	C02572	Level 1	2,135.18
10	*Tyrosol*	1.15	121.0649	C8H10O2	97.9	POS	C06044	Level 2	307.34
11	*Sinapyl alcohol*	3.29	193.0858	C11H14O4	96.5	POS	C02325	Level 2	170.60
12	*Eugenol*	7.42	165.9911	C10H12O2	78.8	POS	C10453	Level 2	76.83
13	*6-Gingerol*	5.07	293.1783	C17H26O4	82.8	NEG	C10462	Level 1	184.77
14	*Zingerone*	7.41	192.9287	C11H14O3	82.0	NEG	C17497	Level 2	35.37
**Flavonoids and glycosides**									
15	*Hesperidin*	3.81	611.1983	C28H34O15	98.4	POS	C09755	Level 1	132.22
16	*Homoeriodictyol*	3.39	177.0542	C16H14O6	98.4	POS	C09756	Level 2	442.17
17	*Wogonin*	4.99	285.0749	C16H12O5	92.3	POS	C10197	Level 1	114.26
18	*Dihydrobiochanin A*	4.98	287.0917	C16H14O5	91.0	POS	C02675	Level 2	112.80
19	*Narirutin*	3.74	581.1834	C27H32O14	94.5	POS	C09793	Level 1	86.01
20	*Poncirin*	4.25	595.2012	C28H34O14	95.3	POS	C09830	Level 1	63.20
21	*Neodiosmin*	3.77	607.1688	C28H32O15	91.6	NEG	HMDB0039856	Level 1	54.80
**Coumarins and derivatives**									
22	*5-Methoxypsoralen*	0.64	215.0332	C12H8O4	84.6	NEG	C01557	Level 2	5,545.84
23	*Sphondin*	7.46	197.8085	C12H8O4	92.4	NEG	C18081	Level 2	661.34
24	*Aesculin*	3.51	177.0192	C15H16O9	92.8	NEG	C09264	Level 1	93.98
25	*Coumarin (glycoside)*	2.99	515.1426	C22H28O14	70.7	NEG	C05851	Level 2	46.61
26	*Scopoletin*	7.39	190.9284	C10H8O4	90.7	NEG	C01752	Level 2	23.35
27	*Decursinol*	0.70	247.2010	C14H14O4	61.1	POS	C09259	Level 2	8.68
28	*Imperatorin*	2.43	292.9980	C16H14O4	61.6	POS	C09269	Level 2	0.60
29	*Pimpinellin*	4.83	245.0493	C13H10O5	72.5	NEG	C09285	Level 1	0.59
**Sterols and vitamins**								Sterols and vitamins	Sterols and vitamins
30	*Cholesterol*	6.65	369.3508	C27H46O	82.7	POS	C00187	Level 2	100.26
31	*Pantothenate*	2.32	220.1178	C9H17NO5	99.8	POS	C00864	Level 1	152.57
**Free amino acids**									
32	*L-Arginine*	0.56	175.1191	C6H14N4O2	99.8	POS	C00062	Level 1	137.29
33	*L-Proline*	0.67	116.0706	C5H9NO2	96.3	POS	C00148	Level 1	12,797.91
34	*L-Threonine*	0.63	120.0654	C4H9NO3	98.1	POS	C00188	Level 2	171.54
35	*L-Tyrosine*	0.97	182.0813	C9H11NO3	96.4	POS	C00082	Level 1	145.22
36	*L-Lysine*	0.55	147.1126	C6H14N2O2	95.6	POS	C00047	Level 1	12.29
37	*L-Histidine*	0.56	156.0768	C6H9N3O2	95.2	POS	C00135	Level 1	35.58
38	*L-Glutamate*	1.20	130.0499	—	94.6	POS	C00025	Level 2	222.69

39	*L-Valine*	0.92	118.0863	—	94.1	POS	C00183	Level 1	790.30
40	*L-Leucine*	1.17	132.1019	C6H13NO2	93.6	POS	C00123	Level 1	4,703.84
41	*L-Isoleucine*	1.10	132.1019	—	93.4	POS	C00407	Level 1	3,938.65
42	*L-Tryptophan*	3.05	203.0830	C11H12N2O2	98.7	NEG	C00078	Level 1	24.77
43	*L-Serine*	0.61	104.0354	—	93.1	NEG	C00065	Level 1	43.48

Level 1: identification confirmed against an authentic reference standard analyzed under identical chromatographic and mass spectrometric conditions (retention time deviation ≤ 0.5 min, together with matching MS1 precursor and MS/MS fragmentation spectra). Level 2: putative annotation based on accurate mass and MS/MS spectral matching to database reference spectra (Total score, column 6), without a corresponding authentic standard. Relative abundance values represent raw MS1 peak areas (arbitrary units, ×10^6) detected in the Phragmites communis rhizoma extract and indicate the relative dominance of each compound within the profiled sample; these are semi-quantitative peak-intensity values and do not represent absolute concentrations.
